# Detection of Norovirus Variant GII.4 Hong Kong in Asia and Europe, 2017−2019

**DOI:** 10.3201/eid2701.203351

**Published:** 2021-01

**Authors:** Martin Chi-Wai Chan, Sunando Roy, Joseph Bonifacio, Lin-Yao Zhang, Preeti Chhabra, Jenny C.M. Chan, Cristina Celma, Mary Ann Igoy, Sin-Leung Lau, Kirran N. Mohammad, Jan Vinjé, Harry Vennema, Judith Breuer, Marion Koopmans, Miranda de Graaf, for NOROPATROL

**Affiliations:** The Chinese University of Hong Kong, Hong Kong, China (M.C.-W. Chan, L.-Y. Zhang, J.C.M. Chan, S.-L. Lau, K.N. Mohammad);; University College London, London, UK (S. Roy, J. Breuer);; Research Institute for Tropical Medicine, Muntinlupa City, the Philippines (J. Bonifacio, M.A. Igoy);; Centers for Disease Control and Prevention, Atlanta, Georgia, USA (P. Chhabra, J. Vinjé);; Public Health England, London (C. Celma);; National Institute for Public Health and the Environment, Bilthoven, the Netherlands (H. Vennema);; Erasmus Medical Center, Rotterdam, the Netherlands (M. Koopmans, M. de Graaf)

**Keywords:** norovirus, viruses, norovirus GII.4, new variant, surveillance, gastroenteritis, enteric infections, communicable diseases, Eurasia, Hong Kong, the Philippines, the Netherlands, United Kingdom

## Abstract

We report a new norovirus GII.4 variant, GII.4 Hong Kong, with low-level circulation in 4 Eurasia countries since mid-2017. Amino acid substitutions in key residues on the virus capsid associated with the emergence of pandemic noroviruses suggest that GII.4 Hong Kong has the potential to become the next pandemic variant.

Noroviruses are a genetically diverse group of RNA viruses found in a plethora of terrestrial, aerial, and aquatic mammalian species, including humans, pigs, cows, sheep, rats, bats, sea lions, and harbor porpoises (*1*). These viruses are classified into 48 genotypes, including the pandemic GII.4 genotype. In humans, norovirus is the cause of almost one fifth of all cases of acute gastroenteritis globally and the leading cause in all age groups (*2*,*3*). The highest disease burden has been documented in young children (*4*,*5*), whereas, in developed countries, infections have been associated with increased deaths in the elderly (*6*). 

Norovirus GII.4 viruses have caused most gastroenteric infections for >2 decades, and new variants have emerged every 2–4 years since 2002 (*7*). Six pandemic GII.4 variants have been named after the place of first reported sequence and year of predominance: US 95–96, Farmington Hills 2002, Hunter 2004, Den Haag 2006, New Orleans 2009, and Sydney 2012. Several norovirus vaccine candidates are in preclinical studies and clinical trials (*8*); however, it is currently unclear if these vaccine formulations could protect against newly emerging GII.4 viruses that are often associated with more norovirus outbreaks and hospitalizations. 

Norovirus surveillance networks, such as NoroNet, CaliciNet U.S. and CaliciNet China, are tracking norovirus genotypes (*9*–*11*). Surveillance data from NoroNet has shown that GII.4 variants can be detected at low levels years before they cause a new pandemic (*9*). We report the detection of a new norovirus variant called GII.4 Hong Kong that was circulating sporadically in communities of 2 countries in Asia and 2 in Europe during 2017–2019. This new variant has sequence features of a potential pandemic GII.4 variant.

## The Study

We recently reported the full genome of a new norovirus GII.4 variant called GII.4 Hong Kong, discovered from the local molecular surveillance of norovirus gastroenteritis in hospitalized patients in Hong Kong, China (*12*). The strain Hu/HK/2019/GII.4 Hong Kong[P31]/CUHK-NS-2200 was collected in August 2019. Preliminary analysis indicated that the full genome had 91.1% nucleotide identity to that of the prototype of the most recent pandemic GII.4 Sydney variant, Hu/GII.4/Sydney/NSW0514/2012/AU, which had predominated worldwide since 2012. The major capsid protein of GII.4 Hong Kong had 89.6% amino acid identity with GII.4 Sydney 2012; phylogenetic distance within this new variant had no overlap with distance between other known GII.4 variants, suggesting a new GII.4 variant (Appendix Figure). Coordinated through the international norovirus classification working group (*1*), the 2 most widely used norovirus and calicivirus typing tools hosted by the National Institute for Public Health (RIVM) of the Netherlands (https://www.rivm.nl/mpf/typingtool/norovirus) and the US Centers for Disease Control and Prevention (https://norovirus.ng.philab.cdc.gov) have been updated and synchronized to include the new norovirus to enable retrospective identification and prospective monitoring of GII.4 Hong Kong.

We subsequently identified and sequenced 4 additional GII.4 Hong Kong strains from archived stool samples from Hong Kong, the Philippines, the Netherlands, and the United Kingdom (Table 1); these strains were collected during May 2017–March 2019 (2 in 2017, 1 in 2018, and 1 in 2019). Prevalence of the new variant was <1% in each country, except the Philippines (Table 1). The youngest patient was an 11-month-old hospitalized infant and the oldest patient was a 90-year-old woman living in a nursing home. 

**Table 1:** Norovirus GII.4 Hong Kong strains detected during 2017–2019

Collection date	Strain designation	City and country of origin	Settings	Patient age/sex	Sequence availability	GenBank accession no.
2017 May	RS17–1713	Pangasinan, Philippines	Inpatient*	11 mo/M	Complete capsid	MT774555
2017 Nov	Zandvoort/0471	Zandvoort, the Netherlands	Sporadic†	23 mo/F	Full genome	MT735394
2018 Mar	CUHK-NS-1772	Hong Kong, China	Inpatient‡	15 y/M	Partial polymerase/capsid	MT577843
2019 Mar	WT_NORO_0887	Manchester, UK	Nursing home§	90 y/F	Full genome	MT742777
2019 Aug	CUHK-NS-2200	Hong Kong, China	Inpatient¶	42 y/F	Full genome	MN400355#

*Eight (12.1%) of 66 genotyped norovirus samples tested positive for GII.4 Hong Kong in 2017 in Philippines. †One (0.2%) of 482 genotyped norovirus samples tested positive for GII.4 Hong Kong in the 2017–18 season in the Netherlands. ‡One (0.3%) of 307 genotyped norovirus samples tested positive for GII.4 Hong Kong in 2018 in Hong Kong. §One (1.0%) of 105 genotyped norovirus samples tested positive for GII.4 Hong Kong in the 2018–19 season in the United Kingdom. ¶One (0.3%) of 368 genotyped norovirus samples tested positive for GII.4 Hong Kong in 2019 in Hong Kong. #Prototype sequence of the GII.4 Hong Kong variant.

We obtained full genomes from 2 patients using next-generation sequencing and a complete major capsid protein sequence from 1 patient using Sanger sequencing. We sequenced part of the polymerase-capsid junction region in another case but could not sequence the complete capsid because of low viral load. All consensus virus sequences have been deposited into GenBank (Table 1). The new GII.4 variant was not found in norovirus outbreaks in the United States.

**Figure 1:**
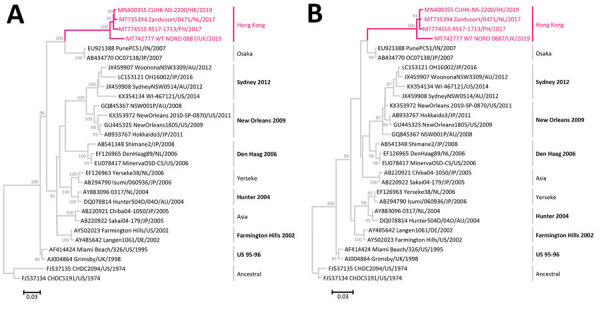
Maximum-likelihood phylogeny of complete sequences of the major capsid protein of norovirus GII.4 variants. A) Nucleotide phylogenetic inference was computed using the Tamura-Nei model with gamma distribution of evolutionary rates among sites. A total of 1,617 positions were included in the final dataset. B) Amino acid phylogenetic inference was computed using the Jones-Taylor-Thornton model with gamma distribution of evolutionary rates among sites. A total of 536 positions were included in the final dataset. Best substitution models were selected using the lowest Bayesian Information Criterion scores. Magenta text indicates the 4 GII.4 Hong Kong sequences. Other GII.4 sequences used as references in the human calicivirus typing tool (https://norovirus.ng.philab.cdc.gov) were downloaded from GenBank. Sequence names are in the following format: GenBank accession no., virus strain name, 2-letter code of country/city of collection, year of collection. Bootstrap values >70% (of 100 iterations) are shown at nodes. Tree branches are drawn to scale; scale bars indicate number of substitutions per site. Trees are rooted to the oldest sequences collected from 1970s. GII.4 variants with pandemic spread are shown in bold text and annotated with the year of predominance (e.g., Sydney 2012); those without pandemic spread are labeled with variant names only (e.g., Osaka). AU, Australia; DE, Germany; HK, Hong Kong; IN, India; JP, Japan; NL, the Netherlands; PH, Philippines; UK, United Kingdom; US, United States.

**Figure 2:**
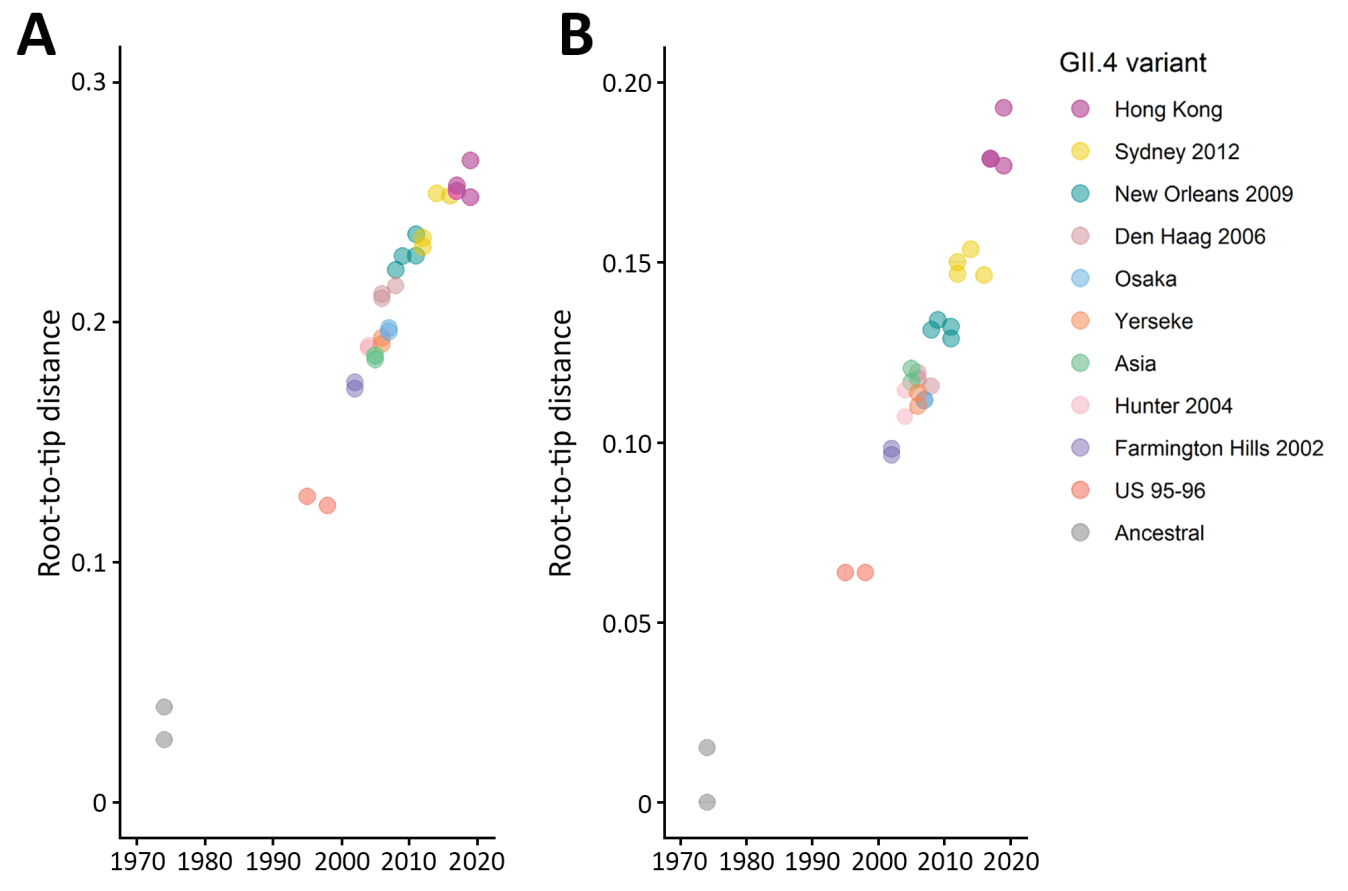
Root-to-tip distance plots of the major capsid protein nucleotide (A) and amino acid (B) sequences of norovirus GII.4 variants. Distance from best-fitting root was calculated using the corresponding maximum-likelihood phylogenetic tree shown in Figure 1. Each circle represents 1 strain color-coded by GII.4 variant; darker shades of color indicate >2 strains of the same variant. R^2^ values indicate the linearity of the accumulation of virus mutations over time; for nucleotide sequences, R^2^ = 0.9691, and for amino acid sequences, R^2^ = 0.9362. An identical set of sequences were used in phylogenetic inference and root-to-tip distance estimation.

We performed sequence analysis on the 4 available complete major capsid protein sequences of GII.4 Hong Kong. Sequence alignment showed that they shared a pairwise identity of 96.4%–99.3% at nucleotide level and 97.2%–99.4% at amino acid level. We performed maximum-likelihood phylogenetic inference using MEGA version 6.06 (http://megasoftware.net). The 4 GII.4 Hong Kong strains formed a monophyletic cluster that was distant from other pandemic GII.4 variants but was closest to GII.4 Osaka, which had no pandemic spread (Figure 1). We calculated root-to-tip distance that reflected phylogenetic relatedness using TempEst version 1.5.3 (http://tree.bio.ed.ac.uk/software/tempest). Sequences of GII.4 Hong Kong had the longest distance from the root at both nucleotide and amino acid levels (Figure 2), indicating the virus had evolved farthest from ancestral GII.4. Inclusion of the new variant preserved and extended the strong linear clock−like relationship of evolutionary distance with time (nucleotide R^2^ = 0.9691; amino acid R^2^ = 0.9362). R^2^ values of ≈1 indicate a nearly constant rate of accumulation of virus mutations over time, which has been characteristic in the molecular evolution of the major capsid protein of GII.4 variants since the 1970s (*7*). Of note, amino acid sequences of GII.4 Hong Kong occupied a distinct spatial-temporal area on the plot with no overlap with other GII.4 variants (Figure 2, panel B). A recent large-scale sequence analysis and antibody blockage confirmation study proposed a set of 5 residues on the major capsid protein that are influential in the emergence and replacement of pandemic GII.4 variants since 1995 (*7*). We observed that residues on 4 of these 5 positions on GII.4 Hong Kong have changed compared with the consensus of GII.4 Sydney 2012 and that 3 residues have changed compared with the consensus of GII.4 Osaka (Table 2).

**Table 2:** Amino acids on the major capsid protein at 5 positions proposed to be influential in the emergence and replacement of pandemic norovirus GII.4 variants since 1995*

GII.4 variant	Amino acid position				
	352	355	357	368	378
Hong Kong	S	A	D	G	G
Osaka	L	S	D	A	G
Sydney 2012	Y	S	D	E	N
New Orleans 2009	Y	S	D	A	N
Den Haag 2006	Y	S	P	S	H
Hunter 2004	S	S	H	S	G
Farmington Hills 2002	S	D	H	N	G
US 1995–96	S	S	H	T	G

*GII.4 variants with pandemic spread are annotated with the year of predominance (e.g., Sydney 2012). Amino acids are shown by 1-letter codes and color-coded to show variations. Source: (*7*).

## Conclusions

The last GII.4 variant, GII.4 Sydney, which has been circulating since 2012, had only a few substitutions when a new recombinant, GII.4 Sydney[P16], emerged in 2015 (*10*). Our findings in 4 countries of low-level circulation (but probably not unique cases) of a new GII.4 variant over a 2-year period clearly indicates that the evolution of GII.4 has been ongoing, if mostly underreported. Phylogeny indicates that the major capsid protein of GII.4 Hong Kong may have evolved from the older GII.4 Osaka variant that was epidemic in 2007, rather than from the newer GII.4 Sydney 2012 variant. The cause of this finding and its implications into GII.4 evolution remain elusive. Although substitutions were found in most of key antigenic residues that have been suggested to be influential in the emergence of new pandemic GII.4 variants, GII.4 Hong Kong has yet to cause outbreaks. Previously, GII.4 variants have been detected at low levels for as long as 18 years before they became pandemic (*13*). GII.4 Hong Kong viruses may still need to further explore the antigenicity landscape to evade herd immunity in humans or to wait for waning of cross-reacting host immunity against GII.4 variants, ultimately allowing pandemic spread of the new variant (*14*). The mechanism for GII.4 viruses to become pandemic remains poorly understood; viral RNA polymerase may also contribute to virus fitness by influencing replication and shedding amount (*15*). Not all GII.4 variants have resulted in pandemics; those of GII.4 Asia, GII.4 Yerseke, and GII.4 Osaka did not. 

In conclusion, we report a new norovirus GII.4 variant called GII.4 Hong Kong that has been circulating sporadically in Eurasia since mid-2017. Because some GII.4 variants do not become predominant pandemic strains, continued surveillance of both outbreaks and sporadic cases is important for monitoring emergent norovirus strains.

Appendix Additional information about norovirus variant GII.4 Hong Kong in Asia and Europe, 2017−2019.

## References

[1] Chhabra P, de Graaf M, Parra GI, Chan MC, Green K, Martella V, et al. Updated classification of norovirus genogroups and genotypes. J Gen Virol. 2019;100:1393–406. 10.1099/jgv.0.001318 31483239 PMC7011714

[2] Havelaar AH, Kirk MD, Torgerson PR, Gibb HJ, Hald T, Lake RJ, et al.; World Health Organization Foodborne Disease Burden Epidemiology Reference Group. World Health Organization global estimates and regional comparisons of the burden of foodborne disease in 2010. PLoS Med. 2015;12:e1001923. 10.1371/journal.pmed.1001923 26633896 PMC4668832

[3] Ahmed SM, Hall AJ, Robinson AE, Verhoef L, Premkumar P, Parashar UD, et al. Global prevalence of norovirus in cases of gastroenteritis: a systematic review and meta-analysis. Lancet Infect Dis. 2014;14:725–30. 10.1016/S1473-3099(14)70767-4 24981041 PMC8006533

[4] Kowalzik F, Riera-Montes M, Verstraeten T, Zepp F. The burden of norovirus disease in children in the European Union. Pediatr Infect Dis J. 2015;34:229–34. 10.1097/INF.0000000000000546 25742072 PMC4338478

[5] Zhou H, Wang S, von Seidlein L, Wang X. The epidemiology of norovirus gastroenteritis in China: disease burden and distribution of genotypes. Front Med. 2020;14:1–7. 10.1007/s11684-019-0733-5 31823287 PMC8320309

[6] Trivedi TK, Desai R, Hall AJ, Patel M, Parashar UD, Lopman BA. Clinical characteristics of norovirus-associated deaths: a systematic literature review. Am J Infect Control. 2013;41:654–7. 10.1016/j.ajic.2012.08.002 23266383

[7] Tohma K, Lepore CJ, Gao Y, Ford-Siltz LA, Parra GI. Population genomics of GII.4 noroviruses reveal complex diversification and new antigenic sites involved in the emergence of pandemic strains. MBio. 2019;10:e02202–19. 10.1128/mBio.02202-19 31551337 PMC6759766

[8] Cates JE, Vinjé J, Parashar U, Hall AJ. Recent advances in human norovirus research and implications for candidate vaccines. Expert Rev Vaccines. 2020;19:539–48. 10.1080/14760584.2020.1777860 32500763 PMC10760411

[9] van Beek J, de Graaf M, Al-Hello H, Allen DJ, Ambert-Balay K, Botteldoorn N, et al.; NoroNet. Molecular surveillance of norovirus, 2005-16: an epidemiological analysis of data collected from the NoroNet network. Lancet Infect Dis. 2018;18:545–53. 10.1016/S1473-3099(18)30059-8 29396001

[10] Cannon JL, Barclay L, Collins NR, Wikswo ME, Castro CJ, Magaña LC, et al. Genetic and epidemiologic trends of norovirus outbreaks in the United States from 2013 to 2016 demonstrated emergence of novel GII.4 recombinant viruses. J Clin Microbiol. 2017;55:2208–21. 10.1128/JCM.00455-17 28490488 PMC5483924

[11] Jin M, Wu S, Kong X, Xie H, Fu J, He Y, et al. Norovirus outbreak surveillance, China, 2016–2018. Emerg Infect Dis. 2020;26:437–45. 10.3201/eid2603.191183 32091361 PMC7045832

[12] Tse EHY, Zhang LY, Lau SL, Chan MC. Genome sequence of a human norovirus GII.4 Hong Kong[P31] variant in Hong Kong, China. Microbiol Resour Announc. 2020;9:e01391–19. 10.1128/MRA.01391-19 31948964 PMC6965582

[13] Allen DJ, Trainor E, Callaghan A, O’Brien SJ, Cunliffe NA, Iturriza-Gómara M. Early detection of epidemic GII-4 norovirus strains in UK and Malawi: role of surveillance of sporadic acute gastroenteritis in anticipating global epidemics. PLoS One. 2016;11:e0146972. 10.1371/journal.pone.0146972 27115152 PMC4846118

[14] Ruis C, Lindesmith LC, Mallory ML, Brewer-Jensen PD, Bryant JM, Costantini V, et al. Preadaptation of pandemic GII.4 noroviruses in unsampled virus reservoirs years before emergence. Virus Evol. 2020;6:veaa067; Epub ahead of print. 10.1093/ve/veaa067 33381305 PMC7751145

[15] Parra GI. Emergence of norovirus strains: A tale of two genes. Virus Evol. 2019;5:vez048. 10.1093/ve/vez048 32161666 PMC6875644

