# Supplementary material for: Detection of Norovirus Variant GII.4 Hong Kong in Asia and Europe, 2017−2019
Source: Emerg Infect Dis. 2021 Jan;27(1):289–93. doi: 10.3201/eid2701.203351 (PMC7774557; doi:10.3201/eid2701.203351)
Supplement: Appendix — Additional information about norovirus variant GII.4 Hong Kong in Asia and Europe, 2017−2019. [file 20-3351-Techapp-s1.pdf]

Appendix DOI: https://doi.org/10.3201/eid2701.203351

Detection of Norovirus Variant GII.4 Hong Kong in Asia and Europe, 2017−2019

Appendix

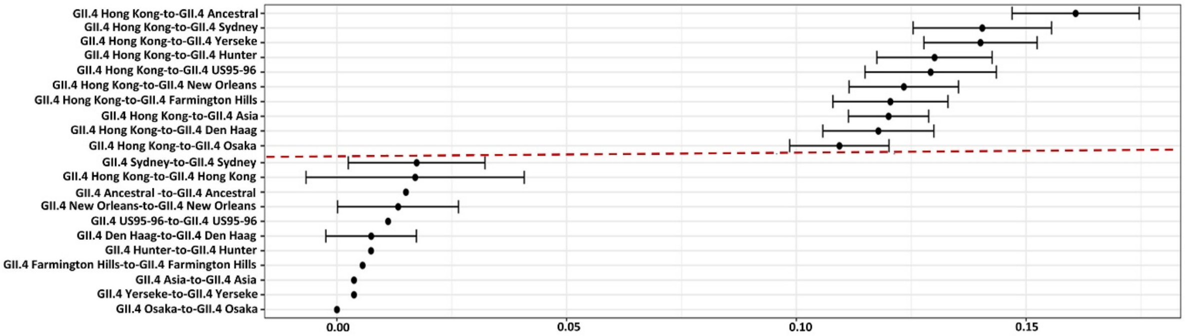

Appendix Figure. Phylogenetic distance comparison of GII.4 Hong Kong viruses with known GII.4 variants (Kroneman et al., 2013). Distances within each GII.4 variant type are shown below the dotted line. Distances between pairs of variant types are shown above the dotted line. Error bars represent 2 SD for each GII.4 variant comparison. A new GII.4 variant is defined as having intravariant phylogenetic distance that does not overlap with distance between other known GII.4 variants.
